# Fibroblast Growth Factors Stimulate Hair Growth through *β*-Catenin and Shh Expression in C57BL/6 Mice

**DOI:** 10.1155/2015/730139

**Published:** 2015-01-01

**Authors:** Wei-hong Lin, Li-Jun Xiang, Hong-Xue Shi, Jian Zhang, Li-ping Jiang, Ping-tao Cai, Zhen-Lang Lin, Bei-Bei Lin, Yan Huang, Hai-Lin Zhang, Xiao-Bing Fu, Ding-Jiong Guo, Xiao-Kun Li, Xiao-Jie Wang, Jian Xiao

**Affiliations:** ^1^Department of Nurse, The first Affiliated Hospital, Wenzhou Medical University, Wenzhou 325035, China; ^2^Molecular Pharmacology Research Center, School of Pharmacy, Wenzhou Medical University, Wenzhou 325035, China; ^3^School of Nurse, Wenzhou Medical University, Wenzhou 325035, China; ^4^Department of Pediatrics, The Second Affiliated Hospital, Wenzhou Medical University, Wenzhou 325000, China; ^5^Wound Healing and Cell Biology Laboratory, Institute of Basic Medical Science, Chinese PLA General Hospital, Beijing 100853, China; ^6^Department of General Surgery, Cixi People's Hospital, Wenzhou Medical University, Ningbo, China

## Abstract

Growth factors are involved in the regulation of hair morphogenesis and cycle hair growth. The present study sought to investigate the hair growth promoting activities of three approved growth factor drugs, fibroblast growth factor 10 (FGF-10), acidic fibroblast growth factor (FGF-1), and basic fibroblast growth factor (FGF-2), and the mechanism of action. We observed that FGFs promoted hair growth by inducing the anagen phase in telogenic C57BL/6 mice. Specifically, the histomorphometric analysis data indicates that topical application of FGFs induced an earlier anagen phase and prolonged the mature anagen phase, in contrast to the control group. Moreover, the immunohistochemical analysis reveals earlier induction of *β*-catenin and Sonic hedgehog (Shh) in hair follicles of the FGFs-treated group. These results suggest that FGFs promote hair growth by inducing the anagen phase in resting hair follicles and might be a potential hair growth-promoting agent.

## 1. Introduction

Hair is considered accessory structure of the integument along with sebaceous glands, sweat glands, and nails. Hair follicle morphogenesis requires the intricately controlled regulation of apoptosis, proliferation, and differentiation. Hair follicles are miniorgans that, during postnatal life, cycle through periods of anagen (growth phase), catagen (regression phase), and telogen (resting phase) [1, 2]. Hair loss is generally not a life-threatening event, but the number of patients suffering from it has increased dramatically. Meanwhile, hair loss takes impact on social interactions and patients' psychological well beings. To date, there are only two anti-hair loss drugs, finasteride and minoxidil, which have been used in clinical, but the effect of these drugs is limited, transient, and somewhat unpredictable [3]. Therefore, it is urgent to develop novel pharmacological treatments.

Various cytokines and growth factors are involved in the regulation of hair morphogenesis and cycle hair growth. The fibroblast growth factor (FGF) family is composed of 22 members with a wide range of biological functions involved in angiogenesis, embryonic development, cell growth, and tissue repair [4, 5]. The early literature reported that acidic fibroblast growth factor (aFGF or FGF-1) and basic fibroblast growth factor (bFGF or FGF-2) may affect the growth of hair follicles, but people have different conclusion. D.C DL demonstrated that exogenous FGF-1 and FGF-2 interfere with follicle morphogenesis and ultimately suppress the hair cycle [6, 7]. On the other hand, Katsuoka et al. reported that FGF-2 promotes papilla cell proliferation and the increase in the size of hair follicle in mice [8, 9]. The controlled release study also showed that gelatin hydrogel enables FGF-2 to positively act on the hair growth cycle of mice [10, 11]. Recent evidence explored another FGF, named keratinocyte growth factor 2 (KGF-2 or FGF-10), which also significantly stimulated human hair-follicle cell proliferation in organ culture [12]. Moreover, FGF-1 has been identified as a crucial endogenous mediator of normal hair follicle growth, development, and differentiation [13], but KGF is not required for wound healing [14]. Therefore, it is necessary for enhanced* in vivo* efficacy to contrive the administration form of FGF.

It should be noted that FGF-1, FGF-2, and FGF-10 have been approved by SFDA for wound healing, and China is the only country in the world for clinical application of these drugs. Thus, the commercial growth factors were used in this study, the hair growth promoting activity was scientifically proven, and the mechanism of action was investigated.

## 2. Materials and Methods

### 2.1. Materials

The DAB chromogen kit (ZSGB-BIO, Beijing, China) was purchased. Anti-*β*-catenin (rabbit polyclonal antibody, Abcam, UK), anti-FGF9 (rabbit polyclonal antibody, Abcam, UK), and anti-Shh (rabbit polyclonal antibody, Santa Cruz Biotech, Santa Cruz, CA, USA) antibodies were purchased. Hematoxylin (Beyotime Institute of Biotechnology, China) and eosin (Beyotime Institute of Biotechnology, China) were purchased. FGF-1 (Shanghai Wanxing Co.), FGF-2 (Zhuhai Essexbio Co.), and FGF-10 (Anhui Xinhuakun Co.) were applied to the experimental group. The concentration of FGFs was diluted at 500 *μ*g/mL.

### 2.2. Experimental Animals

Healthy C57BL6/N mice (6-week-old, 15 mice per group) were obtained from Laboratory Animals Center of Wenzhou Medical University. All animals were from the Laboratory Animals Center of Wenzhou Medical University and were treated strictly in accordance with international ethical guidelines and the National Institutes of Health Guide concerning the Care and Use of Laboratory Animals. The experiments were carried out with the approval of the Animal Experimentation Ethics Committee of Wenzhou Medical University. Temperature (23 ± 2°C), humidity (35–60%), and photoperiod (12 h light and 12 h darkness cycle) were kept constant.

### 2.3. Experimental Studies with FGFs

60 animals in 4 randomized groups (*n* = 15) were used for the study of hair promoting activity. All animals were shaved using depilatory cream (Veet, USA) at 6 weeks of age, at which all hair follicles were synchronized in the telogen stage. FGF-10, FGF-1, FGF-2 (all 500 *μ*g/mL dissolved in 10 *μ*L normal saline, 5 *μ*g/12 cm^2^ per mouse), or vehicle (100 *μ*L normal saline) was applied topically on dorsal skin of C57BL/6N mice with subcutaneous injection for 14 d. At every 1, 7, 14, and 28 d, three mice of each group were sacrificed to obtain skin specimen. Visible hair growth was recorded at every 1, 7, 14, and 28 d.

### 2.4. Histological Studies

Dorsal skin was excised after topical application with FGFs at the indicated time points. Dorsal skin was maintained in 4% paraformaldehyde at 4°C and embedded in paraffin blocks to obtain longitudinal and transverse section. 5 mm sections were stained with hematoxylin and eosin (H&E). The other skin was stored at −80°C for protein extraction. Digital photomicrographs were taken from representative areas at a fixed magnification of 100x.

### 2.5. Hair Follicle Count

The H&E stained slides were photographed using a digital photomicrograph and all of the images were cropped in a fixed area of 300 pixels width. We counted hair follicles manually in a fixed area (0.09 mm^2^). Digital photomicrographs were taken from representative areas at a fixed magnification of 100x.

### 2.6. Hair Length Determination

Hairs were plucked randomly from shaved dorsal area at 1, 7, 14, 21, and 28 days. After plucking 20 hairs per mouse, we measured the average hair length manually.

### 2.7. Immunohistochemistry

Dorsal skins were stained with anti-*β*-catenin and Sonic hedgehog (Shh) antibodies. Sections were dewaxed and hydrated. To quench endogenous peroxidase activity, deparaffinized sections were pretreated with 3% peroxidase for 10 min. After washing with PBS, the sections were incubated with serum to block nonspecific binding of biotinylated secondary antibody for 30 min and then incubated with anti-*β*-catenin (1 : 200) and Shh (1 : 500) antibodies overnight at 4°C. Slides were incubated with biotinylated secondary antibody for 30 min. After incubating with HRP-streptavidin complex to detect secondary antibody for 30 min, slides were developed until light brown staining was visible with DAB chromogen kit. The immunopositivity in fields was counted for per sections using Image-Pro Plus software (Nikon, Tokyo, Japan).

### 2.8. Statistical Analysis

Results are expressed as mean ± SD. Statistical significance was determined with Student's *t*-test when there were two experimental groups. For more than two groups, statistical evaluation of the data was performed using One-way Analysis-of-variance (ANOVA) test, followed by Dunnett's post hoc test with the values *P* < 0.05 considered significant.

## 3. Results

### 3.1. The Effect of FGFs on Hair Growth

**Figure 1:**
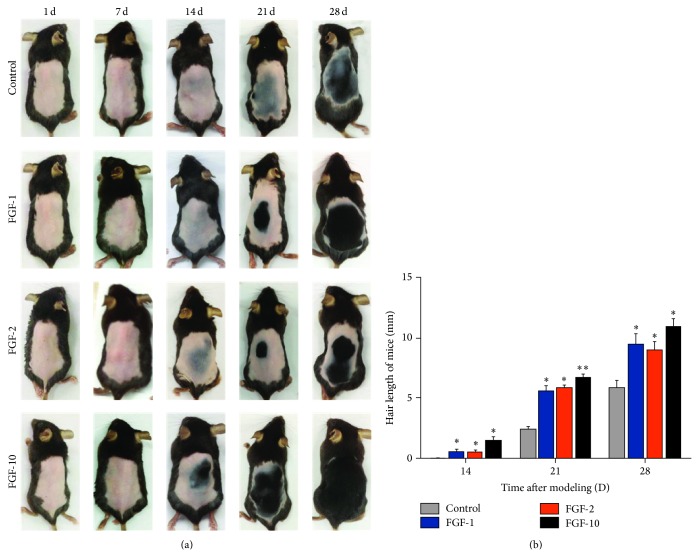
Hair growth promoting effect of FGFs. (a) 6-week-old C57BL6/N mice were shaved and topically applied with vehicle, FGF-1, FGF-2, and FGF-10. Photographs were taken every week after applying FGFs or vehicle on the shaved dorsal skin. (b) Hair length was measured after topical application of FGFs. The hair length of randomly plucked hairs (*n* = 10) was measured at 14, 21, and 28 days after topical application of FGFs. Data shown represent means ± S.D, ^*^ *P* < 0.05, ^**^ *P* < 0.01 versus control group.

The black pigmentation was taken as evidence for transition of hair follicles from telogen to anagen phase. To evaluate the hair growth activity of FGFs, we topically applied FGF-1, FGF-2, and FGF-10 on the shaved dorsal skin of telogenic C57BL/6 mice for 14 d. Each week, we evaluated the degree of hair growth by observing the skin color. At 2 weeks, three FGFs induced black coloration in the shaved skin of C57BL/6 mice significantly; FGF-10 group showed the most black coloration, while very less visible hair growth and black coloration were observed in control group (Figure 1(a)). At 3 weeks, the FGFs group showed markedly hair growth, and FGF-10 stimulated hair growth over 1/2 area on the shaved dorsal skin. At 4 weeks, we observed that hair growth from FGF-1 and FGF-2 was confined to the proximal parts of epidermis; FGF-10 treated group showed overall hair growth which was not confined to the proximal parts. However, the control group only showed less hair growth (Figure 1(a)). To confirm whether FGFs promoted hair growth, we measured the length of 10 hairs plucked from the dorsal skin of each mouse at 2, 3, and 4 weeks. Since visible hair shaft was observed after 2 weeks, we measured length of 2-, 3-, and 4-week-old hairs. As shown in Figure 1(b), the length of hairs in FGF-10, FGF-1, and FGF-2 treated group was remarkably longer than that of control group, and FGF-10 exists with the strongest activity of hair growth. Taken together, these results indicated commercialized drugs, FGF-10, FGF-1, and FGF-2, promote hair growth and FGF-10 appears with the highest efficiency in the three protein drugs.

### 3.2. Effect of FGFs on Hair Follicle Number

**Figure 2:**
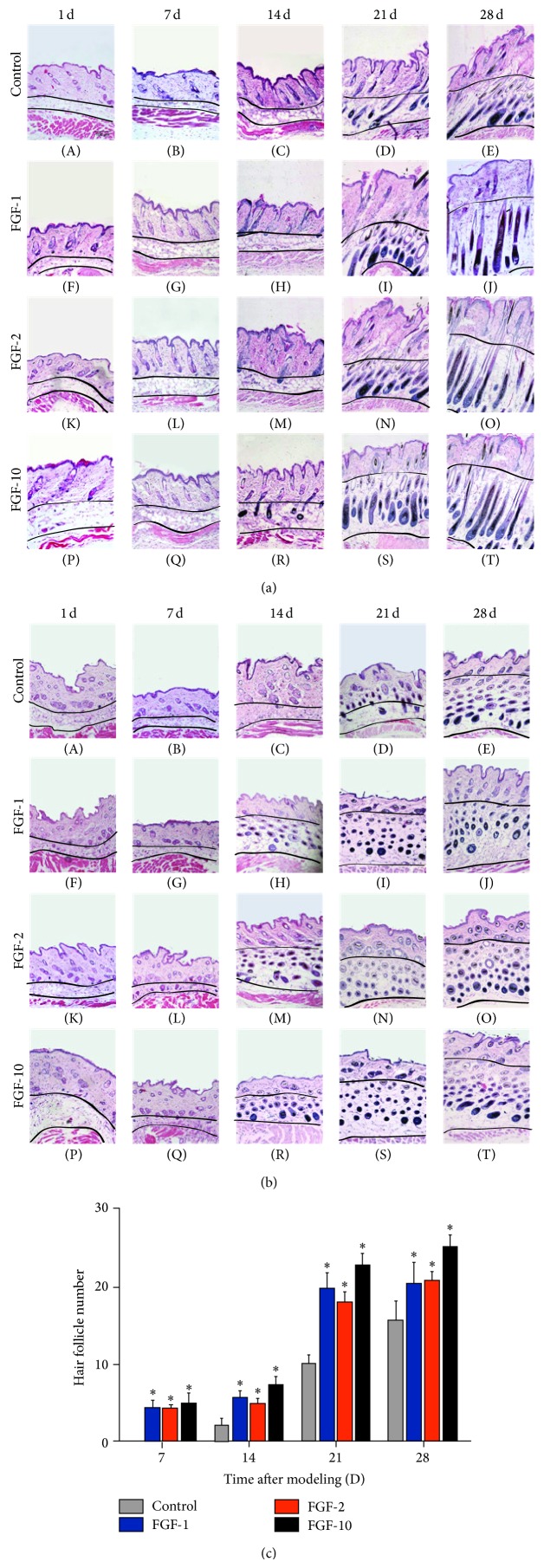
The effect of FGFs on the hair follicles was analyzed by H&E staining. (a) Longitudinal sections of the dorsal skins. (b) Transverse sections of the dorsal skins. (c) The number of hair follicles in deep subcutis. Data shown represent means ± S.D, ^*^ *P* < 0.05, ^**^ *P* < 0.01 versus control group.

It has reported increasing in the number and the size of hair follicles during anagen phase induction [15]. An increase in the density of hair follicles is an indicator for the transition of hair growth from the telogen to anagen phases [16, 17]. To investigate the progression of hair follicles in the hair cycle, HE staining was performed. In the representative longitudinal and transverse sections, the hair follicles in FGF-10, FGF-1, and FGF-2 treated group appeared earlier than those in the control group (Figures 2(a) and 2(b)). Meanwhile, the number of hair follicles of the relative area in FGFs treated group was higher than in the control group, consistent with the above results, and topical application of FGF-10 showed the maximum amount of hair follicles as compared to FGF-1 and FGF-2 group (Figure 2(c)). These data suggested that FGFs including FGF-10, FGF-1, and FGF-2 stimulate hair growth by inducing anagen phase of hair follicles.

### 3.3. FGFs Induced the Expression of *β*-Catenin and Sonic hedgehog (Shh)

**Figure 3:**
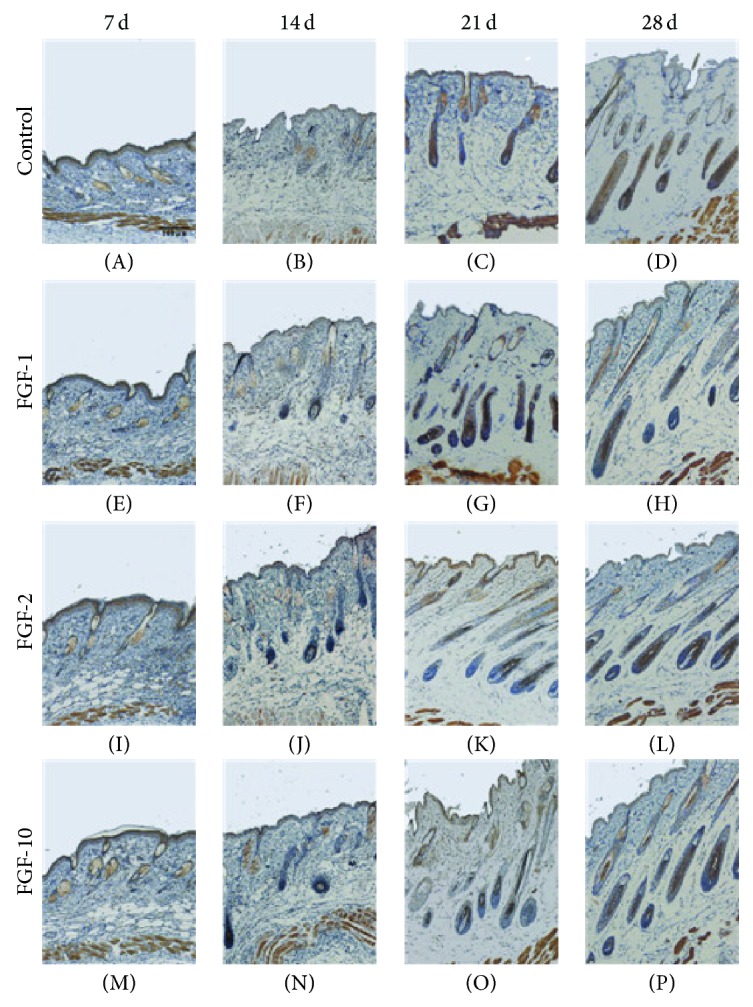
The expression of β-catenin after topical application of FGFs. Longitudinal sections of the dorsal skins from each group were stained for β-catenin by immunohistochemistry (brown staining). Digital photomicrographs were taken from representative areas at a fixed magnification of 100x.

Evidence showed that *β*-catenin induced the transition of the hair growth cycle from the telogen to anagen phases [18, 19]. To elucidate the mechanism of the early events of anagen induction by FGFs, immunohistochemistry analysis was performed to detect the expression of *β*-catenin. We observed that *β*-catenin protein expression appears in FGF-10, FGF-1, and FGF-2 at 1 week, and the levels of *β*-catenin were higher in FGFs group than in control group at 2 weeks (Figure 3). It should be noted that, at 3 weeks, the expression of *β*-catenin was remarkable in control group, while it is decreased in FGF-10 group compared to the appearance at 2 weeks. At 4 weeks, only the control group showed significant expression of *β*-catenin.

**Figure 4:**
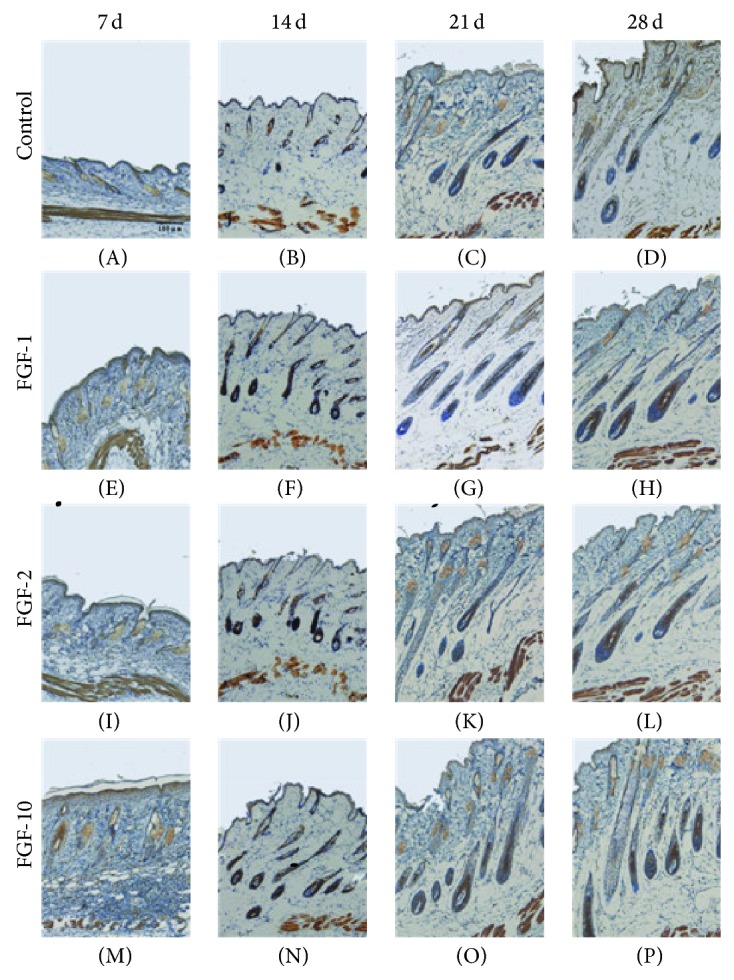
The expression of Shh after topical application of FGFs. Longitudinal sections of the dorsal skins from each group were stained for Shh by immunohistochemistry (brown staining). Digital photomicrographs were taken from representative areas at a fixed magnification of 100x.

The secreted signaling molecule Sonic hedgehog (Shh) plays an important role in both embryonic and adult hair development. In adult mice, Shh expression is upregulated in early anagen, and ectopic application of Shh can prematurely induce anagen in resting telogen follicles [20]. Immunohistochemical analysis result showed that Shh expression was upregulated in FGF-10, FGF-1, and FGF-2 treated group compared to that in control group at 2 weeks (Figure 4). Taking together, these data indicated that the three FGFs drugs promote hair growth partly through upregulating *β*-catenin and Shh.

## 4. Discussion

Hair loss disorders are not life-threatening, but it may make afflicte the people vulnerable and lower their quality of life [21]. The estimated annual market value for hair growth promoting agents is multibillion dollars in the all world. Minoxidil is a widely used hair growth promoting drug for androgenic alopecia patients by inducing hair follicles in the telogen stage to undergo transition into the anagen stages [22]; however, it would also cause adverse dermatological effects, such as dryness, scaling, local irritation, and dermatitis [23, 24]. Finasteride has been reported to be efficacious for androgenic alopecia patients, but it is not recommended for female patients [25]. Therefore, developing new drugs for promoting hair growth is urgently.

Several growth factors (e.g., FGF-1, FGF-2, FGF-7, FGF-10, IGF-1, IGF-2, and EGF) can promote cell cycle and proliferation and have the potential to rescue hair loss and facilitate hair cell regeneration* in vivo* and* in vitro*. It has been shown that EGF and transforming growth factor-*α* (TGF-*α*) are contributed to hair cell proliferation and regeneration in avian utricles [26]. Regeneration of lost hair cells has also been found in rat utricular after treatment with FGF-2 and IGF-1 [27]. KGF protects hair follicles from cell death induced by UV irradiation, chemotherapeutic, or cytotoxic agents [28]. EGF, FGF-1, or FGF-2 maintains high proliferation and multipotent potential of human hair follicle-derived mesenchymal stem cells [29]. All of this literature demonstrated that growth factors may be potential for treating hair loss. Therefore, we searched all of the growth factors which proved by SFDA (China) and we found that FGF-1, FGF-2, and KGF have been applicated in clinical for wound healing, so we purchased these three drugs and investigated the hair growth promoting activity* in vivo*.

C57BL/6 mice are useful models for screening hair growth promoting agents, as their truncal pigmentation is dependent on their follicular melanocytes, producing pigment only during anagen [30]. The shaved back skins of C57BL6/N were treated with topical application of FGFs for 1, 2, 3, and 4 weeks. At 2 weeks, FGF-1, FGF-2, and FGF-10 induced hair growth in the telogenic C57BL/6 mice, while no less visible hair growth was observed in the control group. To further investigate the hair growth promoting effect, we plucked 10 hairs per mouse randomly from the treated area and measured the hair length. The hair length of FGFs treated mice was significantly longer than that of control group. Although FGF-2 is one of the most well-known mitogenic cytokine, interestingly, FGF-2 is not the strongest mitogenic cytokine for hair growth and FGF-10 exerts more potential hair growth promoting effect than FGF-1 and FGF-2. As we know, hair-follicle morphogenesis is governed by epithelial-mesenchymal interactions, between hair placode keratinocytes and fibroblasts of underlying mesenchymal condensations [31]. FGF-10 is found in the dermal papilla fibroblasts and its receptor FGFR2IIIb is found in the neighboring outer root sheath of the keratinocytes [32], suggesting that FGF-10 is a mesenchymally derived stimulator of hair-follicle cells, which contribute to the hair promoting activity.

The Wnt/*β*-catenin pathway plays an important role in the initiation, development, and growth of hair follicles. The transient activation of *β*-catenin results in hair regrowth in mice, while ablation of *β*-catenin results in dramatic hair shortening and abnormal regeneration of hair in the dermal papilla of mouse hair follicles [19, 33]. The levels of *β*-catenin in the dermal papilla are high in the anagen phase but low in the catagen and the telogen phases [18, 34]. Furthermore, the interaction between *β*-catenin, androgen receptors, and keratinocyte growth inhibition through modification of Wnt signaling contributes to androgenic alopecia, a common form of hair loss [35, 36]. Like *β*-catenin, Sonic hedgehog (Shh) also plays a vital role in the morphogenesis of hair follicles and acts as anagen-inducing signaling molecules. Mice lacking Shh activity exhibits follicles arrested at the hair germ stage of development [37, 38]. In the adult, Shh serves as a key regulator to induce the transition from the resting (telogen) to the growth stage (anagen) of the hair follicle cycle [20, 39]. Conversely, antibodies that block the activity of Shh are able to prevent hair growth in adult mice [40]. To elucidate the molecular mechanism underlying the ability of FGFs to induce anagen hair follicles, we examined the protein levels of *β*-catenin and Shh and in the shaved dorsal skin. Our immunohistochemical analysis results showed that the expression levels of *β*-catenin and Shh were upregulated in FGF-10, FGF-1, and FGF-2 treated group compared to that in the control group at 14 days. Its reported continuous *β*-catenin signaling is required to maintain hair follicle tumors [41]; we observed that Shh and *β*-catenin expression levels gradually began to reduce in both groups at 3 and 4 weeks, indicating that anagen phase of hair follicles was ceased.

However, there is no doubt that the limitations of this study still need further investigations and improvements. For example, FGFs are not stable enough which is easy to be degradated by various enzymes* in vitro*, resulting in the loss of biological activity. So the combination with delivery systems to increase its stability may contribute to the functions of promoting hair growth. Moreover, mixtures of several growth factors might to some extent promote cooperation between growth factors and their receptors and contribute together for the protection of hair cells in a sequencing manner or at multiple steps. Further study also should consider the hair growth effect and the mechanism of these FGFs during wound healing. Nevertheless, the effect of FGF-1, FGF-2, and FGF-10 in the therapy of hair loss is confirmative and feasible.

Collectively, our study demonstrated that the commercialized FGF drugs promoted hair growth by inducing anagen in telogenic C57BL6/N mice. FGFs showed significant increase in the number and the size of hair follicles that is considered evidence for anagen phase induction. Immunohistochemical analysis revealed that *β*-catenin and Shh were expressed earlier in FGFs treated group than that in control group. Taken together, these results strongly suggest that FGF-1, FGF-2, and FGF-10 promote hair growth by inducing anagen phase of hair follicles, which is beneficial for the clinical therapy.
